# The Management of a Post-Extraction Gingival Lesion in a Paediatric Patient: A Case Report

**DOI:** 10.3390/children12101331

**Published:** 2025-10-03

**Authors:** Erika Cirillo, Massimiliano Ciribè, Alessandra Putrino, Sonia Vanacore, Francesco Pio Litta, Angela Galeotti

**Affiliations:** 1 Dentistry Unit, Management Innovations, Diagnostics and Clinical Pathways, Bambino Gesù Children’s Hospital, IRCCS, 00165 Rome, Italy; erika.cirillo@opbg.net (E.C.); massimiliano.ciribe@opbg.net (M.C.); alessandra.putrino@opbg.net (A.P.); sonia.vanacore@opbg.net (S.V.); francescopio.litta@opbg.net (F.P.L.); 2 U.N.—E.U. International Research Project on Human Health, Oral Health Section, 1200 Geneva, Switzerland

**Keywords:** gingival lesion, tooth extraction, ozone, child

## Abstract

Introduction: In clinical practice, the presence of abnormal physiological root resorption frequently results in the retention of deciduous teeth. Also, unilateral mastication may contribute to the altered physiological process of root resorption. This delayed exfoliation and retention of deciduous teeth may compromise the integrity of adjacent soft tissue. In recent years, ozone therapy can be considered a promising strategy in accelerating healing and reducing pain in both traumatic and autoimmune ulcers. Case Presentation: This case report describes a 12 year-old male patient with localized damaged gingival tissue resulting from chronic trauma due to the retention of a deciduous tooth. Following the application of gaseous ozone therapy, complete mucosal healing was achieved. Conclusions: This case supports the potential of ozone therapy in paediatric soft tissue management.

## 1. Introduction

During a child’s development, the jaw bones, dentition, periodontium, and the mucogingival complex undergo constant changes. The root resorption (RR) and exfoliation of deciduous teeth (De) represent a complex physiological process that is essential for the normal replacement of deciduous teeth. In normal conditions, RR and De occurs during a specific period of childhood, in order to provide the necessary spatial requirements for the eruption of the permanent successors [1,2]. This process is referred to rizalysis and is characterized by the resorption of hard tissues (dentin and cementum) and the elimination of soft tissues, including the pulp and the periodontal ligament [1].

In clinical practice, the presence of abnormal physiological RR is frequently observed to result in the premature loss or retention of deciduous teeth. Retained deciduous teeth are defined as those that persist beyond the conventional replacement period [1,3].

Unilateral mastication, frequently associated with malocclusions and oral parafunctional habits, has been demonstrated to contribute to the altered physiological process of root resorption, which may result in delayed exfoliation of primary teeth [4,5]. Such functional imbalances can interfere with normal occlusal forces and subsequently affect the timing of tooth exfoliation.

This delayed exfoliation and retention of deciduous teeth may influence the normal development of the permanent dentition and may compromise the integrity of adjacent soft tissue, particularly when the position of the tooth deviates from the conventional eruption pattern [1,6,7,8].

Furthermore, according to the American Academy of Pediatric Dentistry (AAPD), retention of primary teeth can adversely affect the eruption of their permanent successors. Moreover, this condition may contribute to localized periodontal problems. Therefore, it is essential that paediatric patients undergo regular clinical and radiographic monitoring to facilitate early detection and intervention, thereby preventing potential sequelae including soft tissue inflammation and attachment loss [9].

The management of gingival lesions and tissue inflammation in paediatric patients necessitates the implementation of minimally invasive strategies. Chlorhexidine gluconate (CHX) is a frequently used oral healthcare agent due to its antibacterial properties and represent the gold standard for treatment of gingival inflammation [10,11,12,13,14].

Apart from conventional treatment, ozone therapy has emerged as a promising adjunctive agent in medical and dental therapies due to its oxidative and antimicrobial properties [15]: its antibacterial action is primarily attributed to its ability to disrupt bacterial cell walls and stimulate blood circulation by the oxidation of phospholipids and lipoprotein, causing an increase in oxygen release to tissues. When compared with alternative strategies such as topical antiseptics (e.g., chlorhexidine), hydrogen peroxide, systemic antibiotics in selected cases, and photo-biomodulation, ozone therapy offers the advantage of combining antimicrobial efficacy with tissue repair promotion and the absence of common side effects such as staining or taste alteration often associated with chlorhexidine [10,11,12]. Although paediatric-focused systematic reviews remain insufficient, recent meta-analytic evidence confirms its efficacy in enhancing oral mucosal wound healing [16] which reinforces its potential role as a supportive tool also in paediatric patients.

The present article details a clinical case report of a child with a gingival lesion as result of chronic trauma as result of the exfoliation of a deciduous tooth, for the purpose of furthering the understanding of dental professionals in this field.

## 2. Case Report

### 2.1. Patient Information

A 12 year-old male patient in good general health presents in the “Bambino Gesù” Children Hospital (Rome). Growth and development are within normal limits for age. The patient is not under chronic medication and has no history of hospitalizations or surgeries.

### 2.2. Clinical Findings

**Figure 1:**
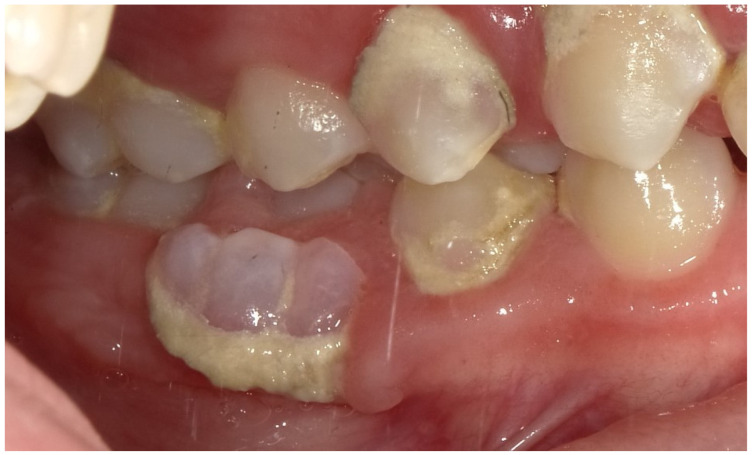
The preoperative intraoral photograph shows the persistence of the lower right primary second molar (#85, FDI system) in a buccal position, associated with a significant calculus deposits in the corresponding quadrants.

**Figure 2:**
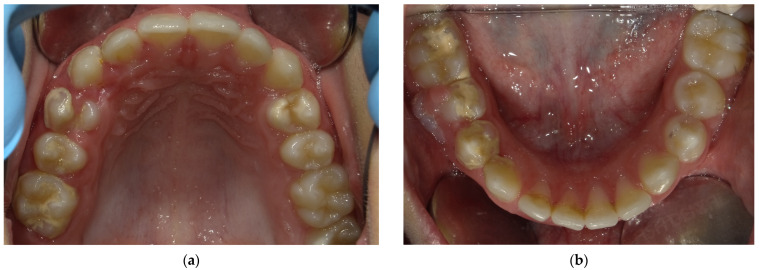
Intraoral occlusal view of the arches: (**a**) The intraoral occlusal view of the maxillary arch shows the presence of calculus deposits in the occlusal area between #16 and #14 with an incomplete eruption of #14. (**b**) The intraoral occlusal view of the mandibular arch shows the presence of calculus deposits in the occlusal area between #46 and #44 and in the lingual area between #31 and #33 with an incomplete eruption of #45.

**Figure 3:**
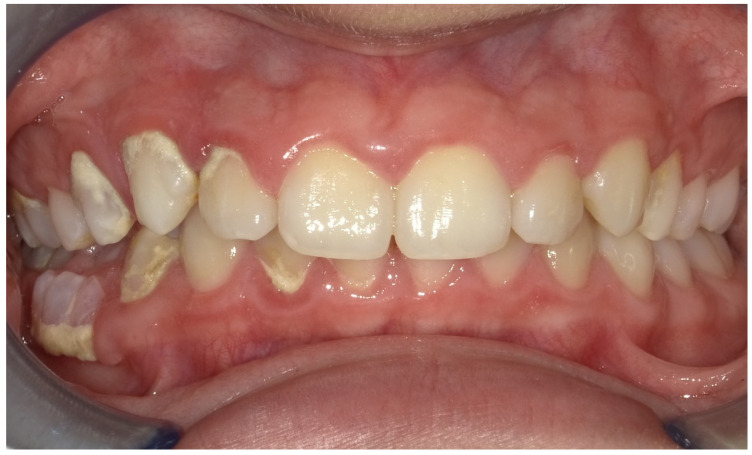
This intraoral frontal view shows an evident supragingival calculus deposits in the first (upper right) and fourth (lower left) quadrants, particularly in the buccal surfaces. The gingival margins in these areas appear inflamed and erythematous.

The patient presents a persistence of lower right primary second molar #85 [17] retained in the buccal region (Figure 1). Moreover, he exhibited an inadequate masticatory pattern, manifesting a left-sided, unilateral chewing movement. This finding is likely attributable to the eruption of permanent teeth and the concomitant discomfort experienced due to the exfoliation of deciduous teeth (Figure 2a,b). This phenomenon led to the accumulation of bacterial plaque and evident calculus formation, due to the absence of the saliva self-cleaning mechanism (Figure 2a,b and Figure 3), and compromised the correct eruption of #14 and #45.

### 2.3. Diagnostic Assessment

Following a comprehensive dental evaluation by the dental team, which included clinical examination, radiographic assessment, and consideration of the patient’s overall oral health status, the dentist made the decision to proceed with the extraction of the retained deciduous tooth due to its persistence beyond the expected exfoliation period, its potential to interfere with the eruption pathway of the permanent successor, and the associated risk of pathological sequelae.

### 2.4. Therapeutic Intervention

Written informed consent was obtained from the patient’s family before the procedure.

Before the extraction procedure, comprehensive oral prophylaxis was carried out by a dental hygienist, involving mechanical removal of both supragingival and subgingival biofilm, as well as calculus deposits. After this procedure, a decontamination of the oral cavity was achieved through rinsing with an antibacterial mouthwash based on 0.2% chlorhexidine digluconate (Curasept ADS|DNA 220 mouthwash (Curasept, Saronno, Italy)). This preoperative decontamination aimed to reduce the microbial burden within the operative field and mitigate the risk of postoperative infectious complication.

Tooth extraction was subsequently carried out using standard operative techniques.

**Figure 4:**
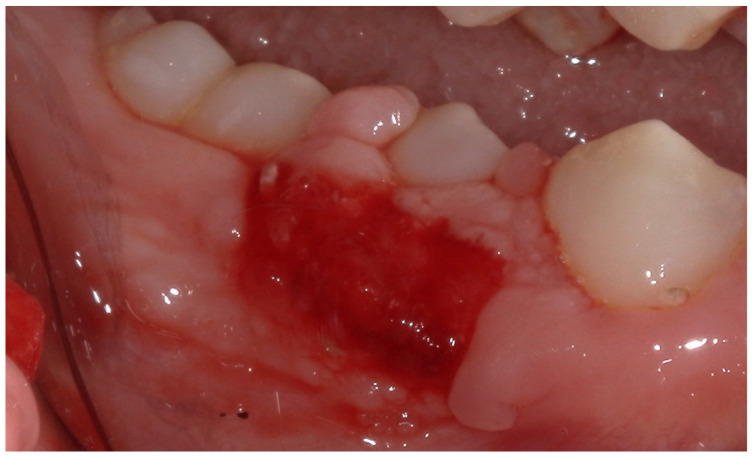
Close-up intraoral view of the extraction site following removal of the retained tooth (#85) and professional oral hygiene. The gingival area of the extraction site appears ulcerated with signs of localized inflammation.

The post-extraction evaluation revealed the presence of damaged gingival tissue, with an ulcerated area (Figure 4) resulting from the chronic trauma due to the vestibular position of the tooth in relation to the physiological eruption site.

Following a detailed evaluation of the patient’s condition, a treatment plan was devised. This comprised three sessions of gaseous ozone therapy administered to the ulcerated area in a total of three sessions over a three-week period (one session of ozone therapy per week). Thus, ozone therapy of soft tissues was performed following the instructions of the ozone generator producer, Ozone DTA—Sweden & Martina (Cornegliana, Italy), which is equipped with integrated safety systems to ensure controlled ozone delivery and operator protection. Ozone was delivered in three sequential cycles, each with a duration ranging from 1 to 2 min, using a progressively increasing power setting (from level 6 to level 8). The primary objective of this intervention was to enhance mucosal healing, prevent secondary infection, and ensure that the patients would not experience any discomfort during their daily functions. At home, the boy was prescribed 0.2% chlorhexidine mouthwash and chlorhexidine gel 1% for the first week (2 rinses a day), and for the last two weeks the patient switched to an ozonated oil-based mouthwash and gel.

### 2.5. Follow-Up and Outcomes

**Figure 5:**
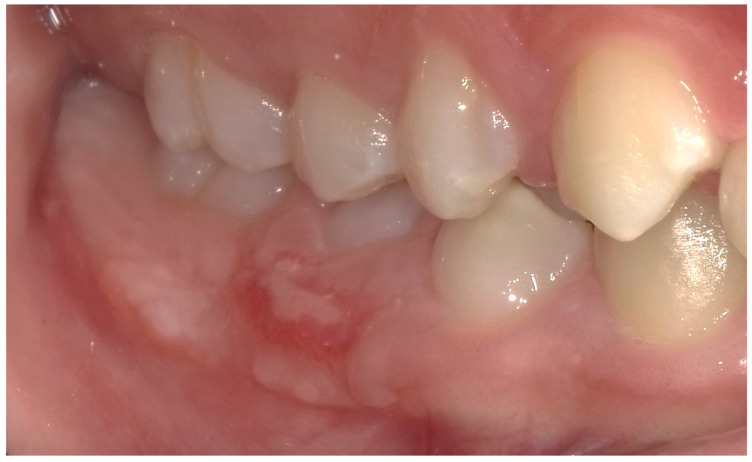
Close-up intraoral view of the gingival lesion during the healing phase, one week after tooth extraction and ozone therapy. The ulcerated area shows partial re-epithelialization and the presence of fibrinous granulation tissue.

**Figure 6:**
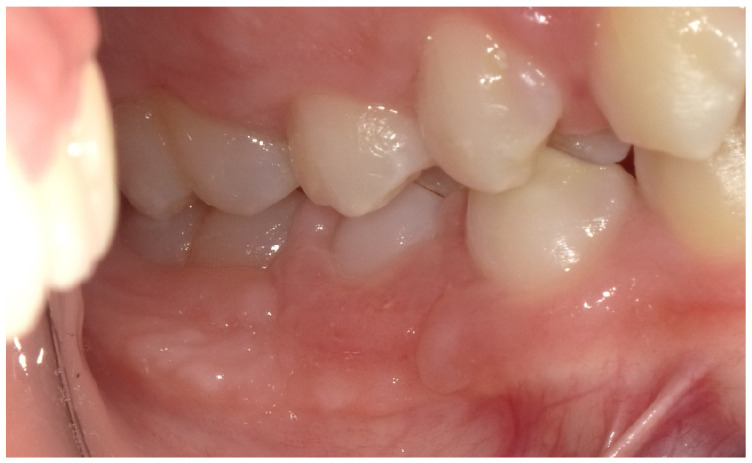
Close-up view of the gingival site two weeks after extraction and completion of ozone therapy sessions. The mucosa appears fully healed, with complete re-epithelialization.

After the first week, a significant decrease in ulceration was observed. Subsequently, fibrinous granulation tissue was evident (Figure 5), and complete mucosal healing was achieved within two weeks (Figure 6).

## 3. Results

The patient responded positively to the proposed treatment protocol.

## 4. Discussion

The physiological replacement of primary teeth is a complex process and a fundamental event that depends on proper root resorption and exfoliation [1]. This process ensures timely exfoliation and eruption of permanent teeth. This mechanism is regulated by various cytokines and transcription factors, such as tumour necrosis factor-α (TNF-α), interleukin-1α (IL-1α), and interleukin-1β (IL-1β) [18,19]. It involves the resorption of hard tissues (dentin, cementum) and elimination of soft tissues (pulp, PDL) [20,21]. Osteoclasts and odontoclasts are responsible for resorbing alveolar bone and root tissues, respectively, and share similar biological behaviour. When this mechanism is interrupted, it can be result in the premature loss or retention of deciduous teeth and associated complications affecting both hard and soft tissues. Issues related to replacement of primary teeth bring conditions like malocclusion, speech problems, and aesthetic concerns [8,22,23,24]. This becomes especially important in cases of conditions that already in themselves impair the possibility of full dentition, such as in the case of agenesis or developmental defects in dental tissues [25,26,27,28].

In the clinical case presented, the retention of a primary tooth led to the development of localized gingival lesions, probably attributable to chronic trauma during a deviated eruption path. This finding is in line with the findings of previous studies, suggesting that altered functional habits (such as unilateral chewing) may interfere with the timing and sequence of physiologic root resorption, consequently inducing altered exfoliation [4,5].

The retention of deciduous teeth beyond the expected exfoliation period has been demonstrated to have a negative effect on the developing permanent dentition [1,6,7,8].

Furthermore, as emphasized by the AAPD, the persistent retention of primary teeth can potentially lead to localized periodontal alteration, including inflammation, soft tissue injury, and loss of attachment [9]. The prolonged retention of primary teeth can be associated with delayed eruption of permanent successors, ectopic eruption, or supernumerary teeth [29,30]. These retained teeth can induce chronic local changes, particularly when surrounded by plaque-retentive restorations or mispositioned within the arch. Such scenarios may facilitate the development of low-grade inflammation in the surrounding gingival and periodontal tissues, particularly when compounded by inadequate oral hygiene or microbial colonization in inaccessible areas, as exemplified in the present case.

The gingival inflammation associated with over-retained primary teeth can manifest as erythema, swelling, or localized gingivitis. Furthermore, the mechanical obstruction caused by the retention of primary teeth may alter normal eruption paths, leading to pseudo-pocket formation and facilitating microbial accumulation—potentially escalating the inflammatory response. In cases involving syndromic conditions such as Proteus syndrome, additional skeletal or dental anomalies can exacerbate soft tissue reactions due to asymmetrical jaw growth or delayed dental development [31].

Although inflammation secondary to this retention is often subclinical in early stages, if left untreated it may evolve into chronic inflammatory states that compromise periodontal health and further delay normal exfoliation. Therefore, timely clinical and radiographic monitoring is essential to identify inflammatory signs and determine whether extraction or interceptive treatment is required [32].

In this clinical case, the gingival lesion was managed through a minimally invasive approach combining conventional antiseptic therapy with adjunctive ozone therapy.

Chlorhexidine is an extensively used antiseptic and remains the gold standard for the non-surgical management of gingival inflammation due to its bactericidal and bacteriostatic properties, as well as its high substantivity [10,11,12,13,14]. Furthermore, chlorhexidine showed a dose- and time-dependent cytotoxic effect on stem cells from exfoliated deciduous teeth. At 0.01% CHX, cell proliferation was reduced by ~50%; at higher concentrations (e.g., 0.1%), the reduction approached 90%. Chlorhexidine exposure also impaired mineralization capacity, with more severe inhibition at higher concentrations and longer exposure times. In regenerative dentistry or when preserving dental pulp stem cells, both Chlorhexidine concentration and exposure duration should be carefully minimized to avoid compromising cell viability and function. Alternative disinfectants or gentler protocols may be preferable in contexts where stem cell preservation is critical [33]. Apart from conventional treatments, ozone therapy has been used and widely investigated as an adjunctive treatment of different oral conditions [15,34,35,36,37,38,39]. First of all, some researchers have studied the clinical effect of ozone during non-surgical treatment of periodontitis [20]. According to a recent systematic review and meta-analysis, ozone therapy has been demonstrated to have a positive effect and can be considered a promising strategy in accelerating healing and reducing pain in both traumatic and autoimmune ulcers; however, the quality of evidence supporting these finding is currently limited [16].

Previous experiences presented in the literature compared 20 ppm ozone gel and 1% chlorhexidine gel in 6–12-year-old children. Both treatments significantly reduced plaque index and Streptococcus mutans counts in dental plaque after use. There was no statistically significant difference between the two gels in terms of antibacterial effect or plaque reduction.

Ozone gel may serve as an effective alternative to 1% chlorhexidine gel for reducing dental plaque and S. mutans in paediatric patients. Since chlorhexidine is associated with side effects such as tooth staining and taste alteration, ozone could be preferable in cases requiring long-term plaque control. These findings support further investigation into ozone-based products as a non-staining, child-friendly antimicrobial option in preventive dentistry [40].

In the clinical case reported here, the adjunctive use of ozone contributed to the resolution of the gingival lesion and overall clinical improvement without adverse effects.

Uncomplicated oral soft tissue wounds (e.g., small mucosal lesions) typically achieve clinical re-epithelialization within 7–14 days, while larger surgical sites may require up to 3–4 weeks. These timelines are consistent with periodontal and oral-mucosal healing literature and early-healing scoring systems used up to 4 weeks post-surgery [41,42]. Chlorhexidine rinses or gels reduce bacterial load and early inflammation, but available clinical evidence does not demonstrate a meaningful shortening of the intrinsic healing time beyond the expected 1–2 weeks [41]. Ozone therapy has been reported to accelerate early healing and reduce postoperative morbidity due to its antimicrobial effect and improvements in local microcirculation and immunomodulation. Several clinical studies in oral surgery and implants show earlier epithelial closure or better early-healing scores versus standard care [43,44,45].

While the treatment showed a positive outcome, these findings are limited to this single case report. Further research is necessary to confirm these results. Moreover, we acknowledge that, as a single case report, the findings may not be directly generalizable to routine clinical practice. Factors such as the individual patient’s clinical conditions and the absence of a comparative data. Moreover, the absence of a clinical photograph of the extracted tooth represents a limitation in the visual documentation of the case.

The presented case highlights the clinical relevance of early diagnosis and management of retained deciduous teeth. It also demonstrates the potential for favourable soft tissue healing through ozone therapy. This case supports the potential of ozone therapy in paediatric soft tissue management.

This case introduces an innovative approach that combines ozone therapy alongside conventional treatments. The novelty lies in the use of ozone therapy as an adjunctive treatment, which has not been widely explored in routine clinical practice. Although ozone has been investigated for various applications in dentistry, its use in combination with traditional therapies remains relatively uncommon.

The educational value of this case provides evidence for the potential benefits of ozone therapy in managing complex oral conditions that may not respond adequately to conventional treatments alone. This case suggests that ozone therapy could offer a promising adjunctive for patients with challenging clinical presentations.

## Data Availability

The protocol was registered on OpenScience Framework on 29 July 2025 (DOI 10.17605/OSF.IO/EB84H).
